# Prognostic Value of Combination of Controlling Nutritional Status and Tumor Marker in Patients with Radical Non-Small-Cell Lung Cancer

**DOI:** 10.1155/2022/4764609

**Published:** 2022-09-23

**Authors:** Keru Ma, Hao Wang, Xiangyu Jiang, Chengyuan Fang, Jianqun Ma

**Affiliations:** ^1^Department of Thoracic Surgery, Harbin Medical University Cancer Hospital, Harbin, China; ^2^Department of Gastrointestinal Surgery, Harbin Medical University Cancer Hospital, Harbin, China

## Abstract

**Background:**

Controlling nutritional status (CONUT) and tumor markers are associated with prognosis in patients with non-small-cell lung cancer (NSCLC). This study is aimed at exploring the potential usefulness of T-CONUT, constructed by combining CONUT and tumor markers, for NSCLC patients undergoing radical surgery.

**Methods:**

A total of 483 patients with NSCLC underwent radical surgical resection. The receiver characteristic operating curve (ROC) was used to select the tumor marker with the highest predictive performance, and CONUT was combined with this marker to construct the T-CONUT. The Kaplan–Meier method and log-rank test were used to analyze the overall survival (OS), and chi-square analysis was used to analyze the association between T-CONUT and clinicopathological characteristics. The independent risk factors were analyzed by Cox regression. A nomogram was constructed by R studio. Calibration plots, the *c*-index, and decision curves were evaluated for the performance of the nomogram.

**Results:**

ROC analysis showed that the predictive performance of CYFRA21–1 was better than that of CEA, NSE, and SCC. CYFRA21–1 was selected for combining with CONUT to construct T-CONUT. Elevated T-CONUT indicates poor prognosis of patients. Histological type, pTNM, and T-CONUT are independent risk factors associated with patient prognosis. The areas under the curve of the nomogram for predicting 3- and 5-year OS were 0.760 and 0.761, respectively.

**Conclusion:**

T-CONUT comprising CYFRA21–1 and CONUT can effectively predict the prognosis of NSCLC patients.

## 1. Introduction

Lung cancer (LC) has the second highest incidence of all cancers and is the leading cause of cancer death [1]. The pathological subtypes of LC are classified into non-small-cell lung cancer (NSCLC) and small-cell lung cancer (SCLC), of which NSCLC is the most common pathological type, accounting for 85% of LCs [2]. Despite significant improvements in treatment in recent years, the prognosis for NSCLC remains poor, with a median survival time of only 30.5 months [3]. Accurately predicting the prognosis of NSCLC patients still faces great challenges, and there are even some differences in the prognosis of patients at the same stage [4].

LC is a malignant tumor with a high incidence of malnutrition and cachexia. Malnutrition has been reported in 26% of NSCLC patients, while 46% of patients are at risk [5]. Malnutrition is not only a common problem faced by NSCLC patients at diagnosis but also a frequent sign during chemotherapy and adjuvant therapy. Esophagitis or anorexia caused by chemoradiotherapy may affect the treatment plan and increase the nutritional burden [6, 7]. Malnutrition can adversely affect the prognosis of patients with NSCLC, such as affected quality of life, treatment-resistant, and increased mortality [8, 9]. Therefore, it is necessary to develop fast, simple, and accurate markers to accurately identify the nutritional status of NSCLC patients before surgery, which is of great value for guiding clinical treatment and predicting prognosis.

Controlling nutritional status (CONUT) is calculated from serum albumin (Alb), total cholesterol (TC), and peripheral lymphocyte count (TLC) [10], which relate to a variety of nutritional indicators that can enable quick assessment of nutritional status and allow the prognosis of NSCLC patients to be accurately predicted [11–13]. In addition to directly reflecting nutritional status, CONUT indirectly reflects the activity of tumor lesions. Elevated CONUT is often accompanied by higher levels of tumor markers [14] that help monitor tumor recurrence and metastasis [15, 16]. Increasing evidence confirms that the T-CONUT score, as jointly constructed from CONUT and tumor marker values, can not only provide a comprehensive preoperative nutritional assessment but also be a natural monitor of tumor activity. Researchers have shown its predictive value in colorectal cancer [17]. In addition, the nomogram constructed by tumor markers can effectively predict the prognosis of NSCLC patients [18]. However, there are few studies on T-CONUT in NSCLC, and the decision of whether to construct a nomogram with T-CONUT for better evaluation of the prognosis of NSCLC still needs to be further explored.

As a result, this study compared the predictive value of different tumor markers for the prognosis of NSCLC patients and selected the tumor marker with the highest predictive performance when combined with CONUT to construct T-CONUT. A nomogram was constructed based on the clinicopathological features of the patients.

## 2. Materials and Methods

### 2.1. Patients

This study retrospectively analyzed NSCLC patients who underwent radical surgical resection at the Department of Thoracic Surgery, Cancer Hospital Affiliated to Harbin Medical University, from December 2011 to May 2016. The diagnosis of NSCLC was based on the intraoperative pathological tissue obtained and confirmed by two pathologists. The patients received routine preoperative examinations during hospitalization, including CT, bone scan, electrocardiogram, routine hematology, and tumor markers. The clinicopathological information of patients was stored in the case system of the Cancer Hospital Affiliated to Harbin Medical University, including gender, age, tumor size, tumor location, and pTNM staging. The above content is in line with the eighth edition of the AJCC Staging Manual [19].

The inclusion criteria were as follows: (1) pathologically diagnosed patients with NSCLC; (2) undergo radical surgery; (3) patients without other malignancies.

The exclusion criteria were as follows: (1) preoperative radiotherapy or chemotherapy; (2) treatment with steroids; (3) autoimmune diseases; (4) serious infection; (5) hematological malignancies.

All patients underwent routine review after surgery, including chest CT, abdominal ultrasound, blood tumor markers, superficial lymph node ultrasound, and head MRI.

### 2.2. Hematology Parameters

Patients underwent routine hematology tests one week before surgery. Prognostic nutritional index (PNI) is calculated as peripheral blood lymphocyte count (×10^9^/ml) × 5 + serum albumin value (g/l) [20]. Systemic inflammation score (SIS) was calculated as follows: Alb < 40 g/l and LMR < 4.44 scored 2; Alb ≥ 40 g/l or LMR ≥ 4.44 scored 1; Alb ≥ 40 g/l and LMR ≥ 4.44 scored 0 [21]. CONUT was calculated by Alb and TLC, which was divided into normal (0-1), mild (2-4), moderate (5-8), and severe (9-12, 14).

### 2.3. Construction of T-CONUT

At first, a receiver characteristic operating curve (ROC) of carcinoembryonic antigen (CEA), cytokeratin 19 fragment (CYFRA21–1), neuron specific enolase (NSE), and squamous cell associated antigen (SCC) was established individually for the NSCLC patients. Then, the area under the curve (AUC) was calculated to determine the optimal cutoff values. The values above the cutoff were considered high, otherwise were considered to be low. Based on the tumor markers and the CONUT values, the patients were divided into four groups: group 1—tumor markers and CONUT both below the cutoff point; group 2—tumor markers below but CONUT above the cutoff point; group 3—tumor markers above while CONUT below the cutoff point; and group 4—tumor markers and CONUT both above the cutoff point.

### 2.4. Statistical Analysis

Overall survival (OS) was defined as the follow-up time from the time of operation to the time of death or the last survival. If the patients were alive at the last follow-up, they were included in this study. ROC was used to calculate the AUC. The optimal cut-off value was calculated using the “Youden index.” Kaplan-Meier method with Long-rank was used to analyze survival curves. Cox regression models were used to analyze the calculation of hazard ratios (HRs) and 95% confidence intervals (CIs) and to identify independent risk factors. T-ROC and nomogram were performed by R studio. Calibration plots, decision curve, and *c*-index were used to validate the performance of nomogram. All analyses were performed using SPSS for Windows version 25.0 and R software version 4.1.2 for statistical analysis, *P* < 0.05 was considered statistically significant.

## 3. Results

### 3.1. Patient Characteristics

The study included 483 patients, 281 males and 202 females, with a median age of 58 (range 25-78) and BMI at 23.39 (range 14.69-32.1). Among them, there were 269 at stage I, 81 at stage II, and 133 at stage III, according to the pTNM definition (Table 1).

### 3.2. Accuracy Comparison of Different Prognostic Markers

**Figure 1:**
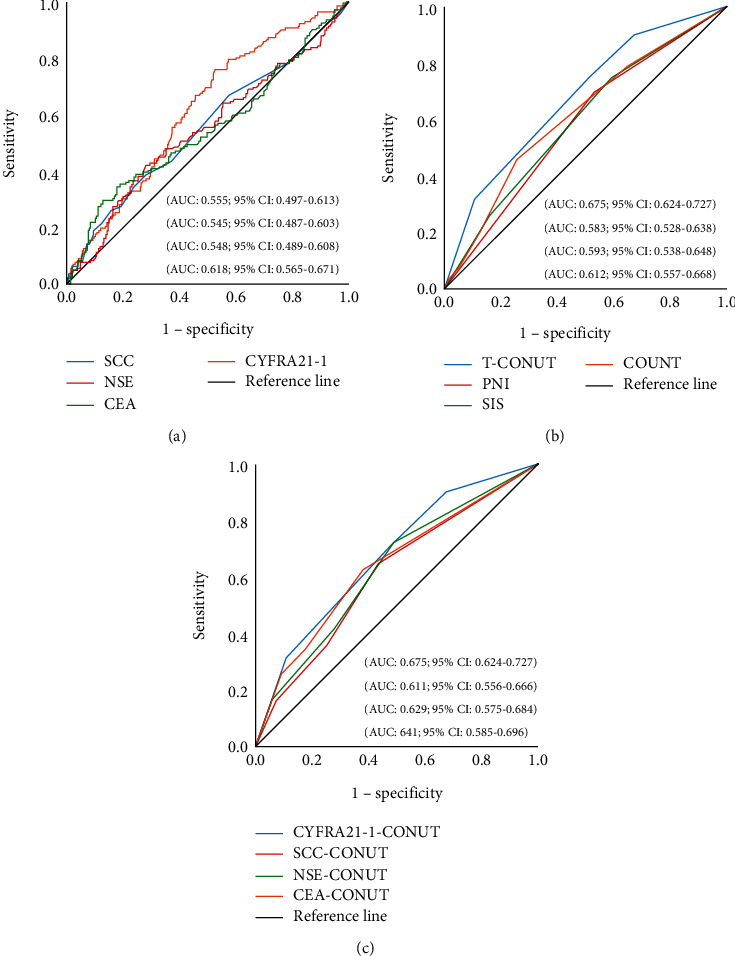
ROC of tumor markers and nutritional markers among total patients. (a) Comparison of predictive performance of different tumor markers. (b) Comparison of predictive performance of T-CONUT with nutritional markers. (c) Comparison of predictive performance of different tumor markers combined with CONUT.

To select tumor markers suitable for evaluating NSCLC according to ROC, CYFRA21-1 and CONUT had the highest AUC (Figures 1(a) and 1(b)), and the cutoff values of CYFRA21-1, CONUT, and PNI are 2.75, 2, and 52.48.

**Figure 2:**
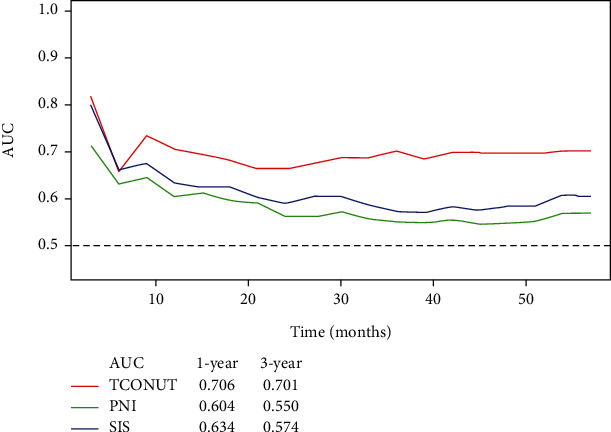
Time-dependent ROC curves for the T-CONUT, PNI, and SIS. The horizontal axis represents month after surgery, and the vertical axis represents the estimated AUC for survival at the time of interest. Red, green, and blue solid lines represent the estimated AUCs for the T-CONUT, PNI, and SIS, respectively.

After finding that CYFRA21-1 and CONUT have the highest AUC, we construct T-CONUT according to the cutoff values of CYFRA21-1 and CONUT. The patients in group 1-group 4 were 125 (25.9%), 71 (14.7%), 205 (42.4%), and 82 (17%), respectively. The prognostic accuracy of T-CONUT, PNI, and SIS was compared by ROC and T-ROC. The results showed that T-CONUT had the highest AUC, which indicated that T-CONUT had high accuracy in predicting OS (Figures 1(b) and 2).

Furthermore, we combine different tumor markers with CONUT and ROC showed that the cutoff values of SCC, NSE, and CEA are 1.15, 14.18, and 6.63. Then, we combine different tumor markers with CONUT, respectively. The AUC of CYFRA21-1-CONUT was higher than that of SCC-CONUT, NSE-CONUT, and CEA-CONUT (Figure 1(c)).

### 3.3. T-CONUT and Patient Survival

Chi-square analysis showed that T-CONUT was associated with sex, SCC, NSE (*P* < 0.001), smoking history, histological type, and pTNM stage and was significantly correlated (Table 2).

**Figure 3:**
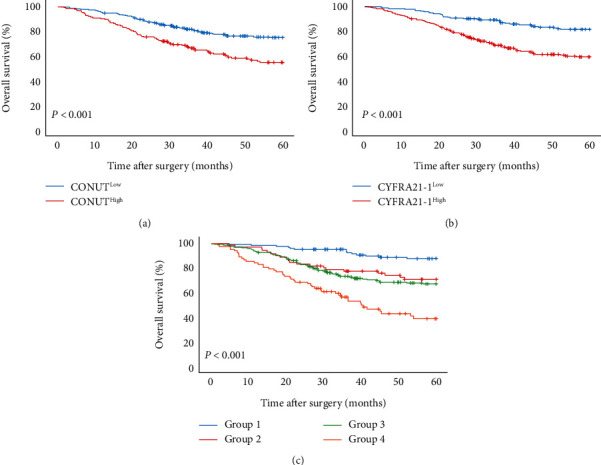
Kaplan–Meier analysis of OS of overall NSCLC patients. (a) Association of the CONUT with the OS of overall patients. (b) Association of the CYFRA21-1 with the OS of overall patients. (c) Association of the T-CONUT with the OS of overall patients.

The survival curve showed that the 5-year OS rate of CONUT ≥ 2 was significantly lower than that of CONUT < 2 (54.5% vs. 74.8%) (Figure 3(a)); the 5-year OS rate of CYFRA21‐1 > 2.75 was significantly lower than that of CYFRA21‐1 ≤ 2.75 (59.0% vs. 81.4%) (Figure 3(b)); for T-CONUT, the 5-year OS rates of group 1-group 4 were 87.6%, 70.7%, 66.8%, and 38.6% (Figure 3(c)). Obviously, elevated T-CONUT indicates poor prognosis of patients.

**Figure 4:**
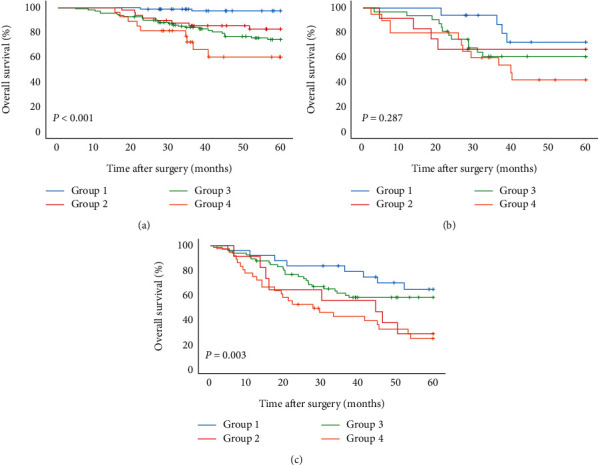
Kaplan–Meier analysis of OS of NSCLC patients at each pTNM stage according to the T-CONUT. (a) Association of the T-CONUT with the OS of patients with stage I NSCLC. (b) Association of the T-CONUT with the OS of patients with stage II NSCLC. (c) Association of the T-CONUT with the OS of patients with stage III NSCLC.

According to pTNM, for stage I, the 5-year OS rates of T-CONUT group 1-4 were 97.4%, 82.7%, 74.3%, and 60.1%. For stage II, the 5-year OS rates of T-CONUT groups 1-4 were 72.4%, 66.7%, 60.8%, and 42.0%. For stage III, the 5-year survival rates of T-CONUT groups 1-4 were 63.8%, 27.3%, 57.2%, and 23.3% (Figures 4(a)–4(c)).

The Cox found that histological type, pTNM, and T-CONUT were independent risk factors (Table 3).

### 3.4. Construction of a Nomogram

**Figure 5:**
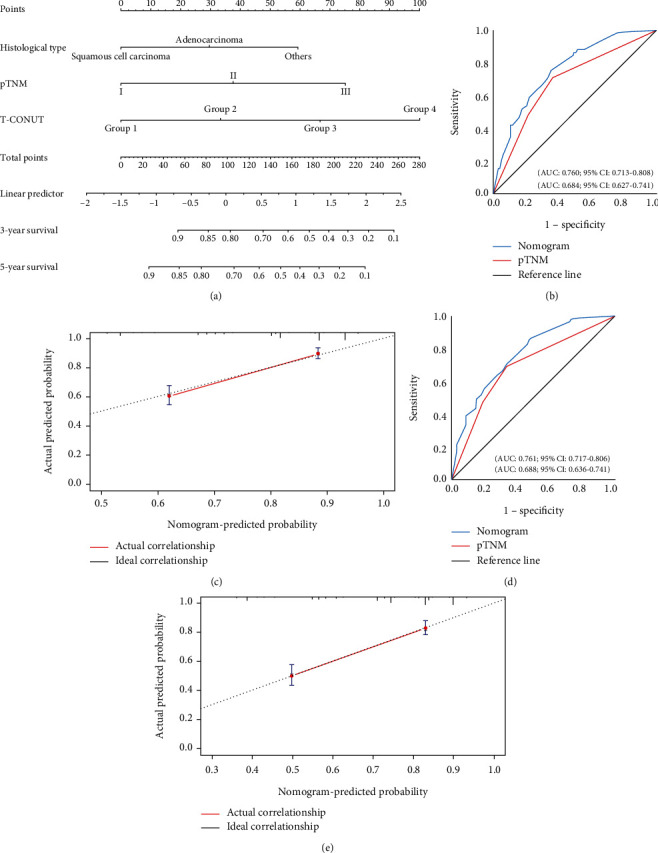
(a) Nomogram model predicting the 3- and 5-year OS of all patients. (b) ROC curve of the nomogram model predicting the 3-year OS of all patients. (c) Calibration curve for 3-year nomogram predictions. (d) ROC curve of the nomogram model predicting the 5-year OS of all patients. (e) Calibration curve for 3-year nomogram predictions.

**Figure 6:**
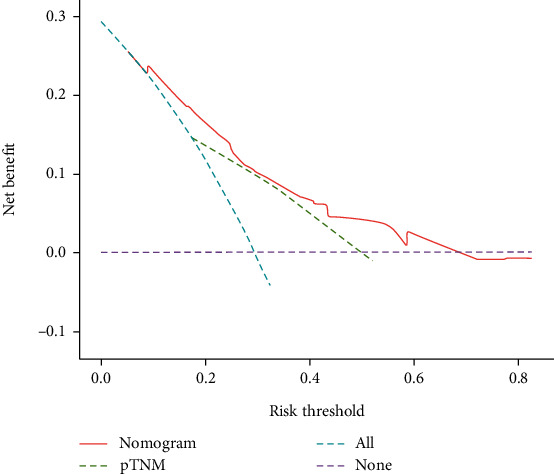
Decision curve analysis for the 5-year survival predictions. In the decision curve analysis, the *y*-axis indicates net beneft, calculated by summing the benefts (true positives) and subtracting the harms (false positives). The nomogram model (red dotted line) had the highest net benefit compared with the pTNM staging system (green dotted line). The straight line represents the assumption that all the patients will die, and the horizontal line represents the assumption that none of the patients will die.

Histological type, pTNM, and T-CONUT were independent risk factors; we combined these factors to construct a nomogram (Figure 5(a)). ROC showed that nomogram had the highest AUC in 3-year and 5-year OS, the sensitivity were 75.5% and 64.6%, and the specificity were 85.2% and 52.5% (Figures 5(b) and 5(d)). The *c*-index was 0.725. Calibration plots and decision curve showed good predictive performance of nomogram (Figures 5(c), 5(f), and 6).

## 4. Discussion

Preoperative nutritional status is crucial for lung cancer patients, and malnutrition adversely affects lung cancer patients, whether for treatment, quality of life, or predicting prognosis. Malnutrition in lung cancer patients may be caused by insufficient intake, impaired function, and complications of adjuvant therapy [22, 23]. A previous study reported that 42.8% of lung cancer patients had malnutrition, a proportion which is significantly higher than for some digestive system tumors such as gastric cancer and colorectal cancer [23]. Therefore, accurately assessing the nutritional status of LC patients is of great significance for better tolerance of adjuvant therapy, nutritional intervention guidance, and prognosis.

In this study, we found that the CONUT score could effectively predict the prognosis of NSCLC patients undergoing radical surgery. CONUT had the highest AUC, which was consistent with previous studies [24]. Part of the reason is that CONUT contains total cholesterol levels, which can reflect inflammation in the body, liver function, changes in body fluids and energy reserves. However, the calculation methods of PNI and SIS do not include total cholesterol. Hikage et al. showed that CONUT has high sensitivity in patients with hypocholesterolemia [25], and changes in total cholesterol levels can effectively judge postoperative recovery and tumor aggressiveness. Total cholesterol levels tend to decrease when tumors recur, suggesting that CONUT can more comprehensively and accurately reflect the nutritional status of NSCLC [26]. In addition, there are certain differences in the nutritional status of ethnic groups in different regions. For example, the obesity rate in Western countries is higher than in Eastern countries, and the obesity rate in economically developed regions is higher than in low-income regions [27–29]. Therefore, some specific nutritional scores help better predict prognosis [30, 31]. Our institution is a high-capacity center in Northeast China, and the results also have certain applicable value. Although our results may have limited clinical applicability, this does not prevent us from making recommendations for LC experts: for LC patients with a high incidence of malnutrition, variations in the nutritional status of lung cancer patients with different physiques in different regions needs to be recognized. At the same time, we also call for multicenter and large-sample studies to use region and ethnicity as independent evaluation factors for nutritional indicators, which will help to more accurately assess the nutritional status of patients before surgery.

We found that elevated CONUT was associated with poor prognosis, suggesting that poor nutritional status will lead to poor prognosis. This finding indicates that different nutritional indicators play different roles in NSCLC patients. Albumin is synthesized by the liver and has antitumor oxidative properties [32]. Decreased albumin reflects a loss of body defenses and reduced responsiveness to antitumor therapy [33]. Guner et al. showed that albumin was more prognostic than NLR and PLR [34], and the score weight of albumin in the CONUT score was twice that of other indicators, which also shows that albumin has importance for the prognosis of patients. Cholesterol participates in the basic structure of cell membranes and maintains cell physiological functions through intracellular signal transduction. Decreased cholesterol levels suggest both a lack of energy storage and metabolic imbalances that contribute to the development and progression of cancer [35]. As an important part of the immune system, lymphocytes inhibit tumor cell growth, invasion, and migration by exerting cytotoxic functions, and their decreased levels suggest a poor prognosis for patients [36]. These mechanisms explain why elevated CONUT is associated with poor patient outcomes.

Notably, some studies have found that CONUT is significantly associated with tumor marker levels, and when CONUT is elevated, it is often accompanied by higher tumor marker levels [14, 37]. Selecting specific tumor markers according to different tumor tissues helps to accurately determine the biological behavior of tumors. The T-CONUT constructed by Yamamoto et al. combines CEA and CONUT and can effectively predict the prognosis of colorectal cancer patients [17]. T-CONUT constructed by Wang et al. as a combination of CA19-9 and CONUT can well predict the prognosis of patients with pancreatic ductal adenocarcinoma [38]. These studies suggest that the combined score can provide more comprehensive prognostic information and better serve patients. They also provide a good theoretical basis for us to combine tumor markers with CONUT as T-CONUT may have better predictive performance for NSCLC.

ROC showed that CYFRA21-1 was more accurate in predicting prognosis than CEA, SCC, and NSE, which was consistent with previous studies [39]. Although the sensitivity of tumor markers depends on tumor tissue classification, different studies have shown that CYFRA21-1 can effectively assess the prognosis of NSCLC patients. Zhang et al. found that CYFRA21-1 was an independent risk factor associated with the prognosis of lung adenocarcinoma whose predictive power was better than that of CEA and CSE [16]. Reinmuth et al. showed that CYFRA21-1 was more sensitive to lung squamous cell carcinoma than CEA, NSE, and SCC [40]. These results suggest that CYFRA21-1 still has good applicability, even for different histological types. In addition, we combined different tumor markers with CONUT and still found that T-CONUT, constructed with CYFRA21-1 and CONUT, had the highest AUC. This indicates that CYFRA21-1-CONUT is more suitable for predicting the prognosis of NSCLC patients; it can provide simple, accurate, and rapid preoperative evaluation and has definite applicable value.

After constructing T-CONUT from CYFRA21-1 and CONUT, we found that strong tumor aggression was associated with increased T-CONUT, suggesting that greater aggression would lead to lower nutritional status. In addition, we also found that when T-CONUT scores were higher, there was a significantly increased proportion of patients who smoked. Smoking is one of the causes of LC. Smoking may suppress appetite and affect eating [41] while anorexia can lead to insufficient nutritional intake and weight loss. As anorexia progresses, lung cancer patients may eventually develop refractory cachexia [42]. Smoking can also lead to more aggressive lung cancer, acquisition of drug resistance, and ultimately, poor prognosis [43, 44]. Therefore, smoking cessation will not only reduce the incidence of LC but also help improve nutritional status.

Clinically, pTNM staging can accurately assess the biological behavior of NSCLC and predict prognosis. However, nutritional status gradually decreases with increasing pTNM staging, and pTNM staging based on macroscopic anatomy cannot provide microscopic blood prognostic information. Some blood nutritional markers, such as albumin serum and total cholesterol, have the advantages of being cheap and easy to apply and can allow rapid preoperative assessment. More importantly, these blood nutritional markers can accurately predict the prognosis of patients with NSCLC. Li et al. constructed a nomogram based on pTNM staging and the albumin-fibrin ratio to accurately predict the prognosis of NSCLC [45]. Guo et al. constructed a combination of N stage and serum albumin to globulin ratio as a nomogram to accurately predict long-term survival in NSCLC [46]. Zeng et al. constructed C-reactive protein and TNM staging as a nomogram which can accurately predict the prognosis of NSCLC patients undergoing radical surgery [47]. These studies demonstrate that predictive models constructed from anatomical staging and nutritional markers can personalize patient risk stratification, reduce prognostic bias, and serve patients better. Notably, we combined different tumor markers with CONUT and ROC and showed that CYFRA21-1-CONUT had the highest AUC. Moreover, Cox analysis showed that T-CONUT constructed from CYFRA21-1 and CONUT, histological type, and pTNM stage were independent risk factors associated with patient prognosis. Afterward, we integrated the above parameters to construct a nomogram model. However, although a nomogram built with CYFRA21-1-CONUT had the highest AUC, the AUC values for ROCs in this study were not as high as expected. We think this may be due to the small sample size, also recognizing that a larger sample will be required for validation. Nevertheless, the AUC of the nomogram in predicting the patient's OS at both 3 and 5 years was higher than that of pTNM staging. This indicates that T-CONUT can supplement pTNM staging with more comprehensive prognostic information. This study suggests that the nomogram constructed with T-CONUT can provide clinicians with more comprehensive tumor prognostic information, which is helpful for comprehensively grasping the biological behavior of the tumor, and its prognostic performance is higher than that of traditional pTNM staging. The calibration plot showed that the nomogram performed well in predicting the status of patients at 3 and 5 years. The advantages of the nomogram also show that both nutritional status and pTNM staging play an important role in predicting the prognosis of NSCLC. Some nutritional markers supplement pTNM staging with more comprehensive prognostic information. The nomogram combined with staging can better serve NSCLC patients and is worthy of clinical application.

### 4.1. Research Limitations

There are some limitations to this retrospective study. First, the results of this study require multicenter validation. Second, we only collected the information of tumor markers and nutritional markers of patients before surgery, and dynamic monitoring of these indicators can help predict prognosis more accurately.

## 5. Conclusion

The T-CONUT constructed by the combination of CYFRA21-1 and CONUT can effectively predict the prognosis of NSCLC patients. An elevated T-CONUT group suggested a poor prognosis. Furthermore, the nomogram constructed by T-CONUT combined with the clinicopathological features of the patients helps to better serve NSCLC patients.

## Figures and Tables

**Table 1:** Patient characteristics.

Clinicopathological features	Patients 483(%)
Sex	
Male	281 (58.2)
Female	202 (41.8)
Age (years)	
≤60	284 (58.8)
>60	199 (41.2)
BMI (kg/m^2^), median, range	23.39 (14.69-32.1)
SCC (ng/ml)	
≤1.5	409 (84.7)
>1.5	74 (15.3)
CEA (ng/ml)	
≤5	327 (67.7)
>5	156 (32.3)
NSE (ng/ml)	
≤15.2	369 (76.4)
>15.2	114 (23.6)
CYFRA21-1 (ng/ml)	
≤3.3	259 (53.6)
>3.3	224 (46.4)
Smoking history	
No	227 (47.0)
Yes	256 (53.0)
Tumor location	
Left lung	192 (39.8)
Right lung	291 (60.2)
Histological type	
Squamous cell carcinoma	157 (32.5)
Adenocarcinoma	301 (62.3)
Others	25 (5.2)
pTNM	
I	269 (55.7)
II	81 (16.8)
III	133 (27.5)
CONUT	
<2	330 (68.3)
≥2	153 (31.7)
PNI	
≥52.48	203 (42.0)
<52.48	280 (58.0)
SIS	
0	175 (36.2)
1	215 (44.5)
2	93 (19.3)

**Table 2:** Chi-square analysis of T-CONUT and patient characteristics.

Clinicopathological features	Group 1 (125)	Group 2 (71)	Group 3 (205)	Group 4 (82)	*P*
Sex					**0.001**
Male	56 (44.8)	41 (57.7)	126 (61.5)	58 (70.7)	
Female	69 (55.2)	30 (42.3)	79 (38.5)	24 (29.3)	
Age (years)					0.209
≤60	78 (62.4)	47 (66.2)	110 (53.7)	49 (59.8)	
>60	47 (37.6)	24 (33.8)	95 (46.3)	33 (40.2)	
BMI (kg/m^2^)					0.093
≤23.39	58 (46.4)	34 (47.9)	96 (46.8)	51 (62.2)	
>23.39	67 (53.6)	37 (52.1)	109 (53.2)	31 (37.8)	
SCC (ng/ml)					**<0.001**
≤1.5	120 (96.0)	65 (91.5)	166 (81.0)	58 (70.7)	
>1.5	5 (4.0)	6 (8.5)	39 (19.0)	24 (29.3)	
CEA (ng/ml)					0.275
≤5	86 (68.8)	49 (69.0)	144 (70.2)	48 (58.5)	
>5	39 (31.2)	22 (31.0)	61 (29.8)	34 (41.5)	
NSE (ng/ml)					**<0.001**
≤15.2	111 (88.8)	67 (94.4)	137 (66.8)	54 (65.9)	
>15.2	14 (11.2)	4 (5.6)	68 (33.2)	28 (34.1)	
Smoking history					**0.003**
No	73 (58.4)	39 (54.9)	80 (39.0)	35 (42.7)	
Yes	52 (41.6)	32 (45.1)	125 (61.0)	47 (57.3)	
Tumor location					0.302
Left lung	51 (40.8)	29 (40.8)	73 (35.6)	39 (47.6)	
Right lung	74 (59.2)	42 (59.2)	132 (64.4)	43 (52.4)	
Histological type					**<0.001**
Squamous cell carcinoma	17 (13.6)	21 (29.6)	88 (42.9)	31 (37.8)	
Adenocarcinoma	105 (84.0)	46 (64.8)	108 (52.7)	42 (51.2)	
Others	3 (2.4)	4 (5.6)	9 (4.4)	9 (11.0)	
pTNM					**<0.001**
I	84 (67.2)	48 (67.6)	110 (53.7)	27 (32.9)	
II	17 (13.6)	12 (16.9)	32 (15.6)	20 (24.4)	
III	24 (19.2)	11 (15.5)	63 (30.7)	35 (42.7)	

**Table 3:** The Cox regression of overall patients.

Clinicopathological features				
	Univariate analysis		Multivariate analysis	
	HR (95% CI)	*P*	HR (95% CI)	*P*
Sex		0.111		
Male	1			
Female	0.757 (0.537-1.066)			
Age	0.994 (0.976-1.014)	0.566		
BMI	0.954 (0.907-1.002)	0.061		
SCC	1.059 (1.004-1.117)	**0.034**	1.015 (0.952-1.081)	0.655
CEA	1.000 (0.997-1.004)	0.811		
NSE	0.999 (0.995-1.004)	0.786		
Smoking history		0.513		
No	1			
Yes	1.117 (0.802-1.555)			
Tumor location		0.484		
Left lung	1			
Right lung	1.130 (0.803-1.589)			
Histological type		**0.014**		**0.004**
Squamous cell carcinoma	1		1	
Adenocarcinoma	1.230 (0.845-1.791)	0.280	1.856 (1.211-2.843)	**0.005**
Others	2.558 (1.364-4.797)	**0.003**	2.513 (1.316-4.801)	**0.005**
pTNM		**<0.001**		**<0.001**
I	1		1	
II	2.806 (1.771-4.445)	**<0.001**	2.614 (1.619-4.222)	**<0.001**
III	4.036 (2.757-5.907)	**<0.001**	3.248 (2.183-4.834)	**<0.001**
T-CONUT		**<0.001**		**<0.001**
0	1		1	
1	2.749 (1.389-5.443)	**0.004**	2.720 (1.352-5.471)	**0.005**
2	3.254 (1.823-5.808)	**<0.001**	3.252 (1.800-5.874)	**<0.001**
3	7.204 (3.948-13.145)	**<0.001**	4.716 (2.470-9.003)	**<0.001**
PNI		**0.002**		0.336
≥52.48	1		1	
<52.48	1.744 (1.219-2.495)		1.243 (0.798-1.938)	
SIS		**0.003**		0.466
0	1		1	
1	1.746 (1.167-2.612)	**0.007**	1.315 (0.840-2.057)	0.231
2	2.175 (1.374-3.442)	**0.001**	1.338 (0.761-2.353)	0.311

## Data Availability

The data used to support the findings of the manuscript are available by contacting the corresponding author upon reasonable request.
